# Complications of dermal filler treatments—a registry-based study from Finland

**DOI:** 10.2340/aos.v85.46216

**Published:** 2026-06-15

**Authors:** Vilma Koivisto, Jaana Rautava, Hanna K. Laine

**Affiliations:** a Department of Oral and Maxillofacial Diseases, Clinicum, University of Helsinki, Helsinki, Finland; b Department of Pathology, HUSLAB, HUS Diagnostic Center, Helsinki University Hospital and University of Helsinki, Helsinki, Finland

**Keywords:** Complication, Finnish authority, reclamation, complaint, dermal fillers

## Abstract

The popularity of dermal filler treatments is increasing in the Nordic countries. In Finland, legislation does not mandate healthcare training for those performing dermal filler treatments, potentially leading to increased complications. This registry-based study aimed to characterize complaints on dermal filler treatments received by Finnish Authorities, using data from (1) Finnish Safety and Chemicals Agency (TUKES), (2) the National Supervisory Authority for Welfare and Health (Valvira), (3) the Finnish Competition and Consumer Authority (KKV), and (4) Regional State Administrative Agency (AVI). From January 1, 2017, to December 31, 2024, 19 complaints were received by KKV, unsuccessful lip filler treatments being the most common issue (59%). No life-threatening complications were reported. Most cases (56%) occurred in beauty salons, with treatments primarily performed by beauty salon employees (44%), followed by registered nurses (24%), and only 8% by medical doctors. Notably, 72% of the complications occurred in the last 3 years. The study underscores the need for regulatory oversight and advanced medical expertise in administering dermal filler treatments due to the increasing complications and the involvement of non-medical personnel. Complications may require immediate medical first aid and should be classified as procedures requiring advanced medical expertise.

## Introduction

Dermal filler treatments have significantly increased in popularity. In 2023, the global market size for dermal fillers was estimated at 8.93 billion US dollars, and it is believed to grow to 17.24 billion by 2032 [1]. The most used filler is hyaluronic acid-based gel [2], which is classified in the European Union (EU) as a Class III medical device with Conformité Européenne (CE) marking [3].

Dermal filler treatments carry a risk of complications, since foreign material is injected via needle under surface of skin or mucosa. These complications can be categorized as immediate or delayed. A severe immediate complication occurs when the filler enters the bloodstream, potentially causing tissue necrosis [4], blindness, or a stroke [5, 6]. Delayed complications, such as infection or foreign body reactions, may result from inadequate aseptic techniques or low-quality filler products [7]. In case of delayed complication of hyaluronic acid-based fillers, the substance could be dissolved with hyaluronidase [8]. In previous studies from Denmark, Netherlands, and the United States, the most common complications have been found to be swelling, nodule, inflammation, granuloma formation, and discoloration [9–11]. These studies are usually conducted by a single center, and prevalence of complications are not available.

In Finland, popularity of dermal filler treatments for esthetics is increasing. Finnish legislation does not demand any healthcare professionalism to act as filler practitioners, unlike neighboring country Sweden, where only licensed healthcare professionals (nurse, dentist, or medical doctor) are permitted to perform these procedures [12]. However in Finland, in case of complication, use of hyaluronidase [8] is restricted to physicians and dentists. The popularity of filler treatments increases the risk of complications but in case of non-healthcare professional providing the treatment, the consumer is not protected by patient insurance [13]. The aim of this registry-based study was to characterize complaints of complications reported to Finnish Authorities on dermal filler treatments.

## Materials and methods

Data for the study were requested from (1) Finnish Safety and Chemicals Agency (TUKES), (2) the National Supervisory Authority for Welfare and Health (Valvira), (3) the Finnish Competition and Consumer Authority (KKV), and (4) Regional State Administrative Agency (AVI). These data are produced without any identifying information and are available for anyone upon request and does not require ethical approval. The data were retrieved from January 1, 2017 to December 31, 2024.

The selection of TUKES, Valvira, KKV, and AVI was based on their distinct roles in overseeing safety and compliance across various sectors in Finland. TUKES is responsible for monitoring the safety and compliance of chemicals, products, services, and industrial activities [14]. Valvira oversees patient rights, healthcare professionals, and healthcare organizations [15]. KKV is tasked with enforcing consumer protection laws, competition laws, and public procurement regulations [16]. AVI supervises companies that provide healthcare services [17]. These authorities were chosen because they are likely the primary points of contact for individuals seeking resolution in the event of complications. Complications that fall under patient insurance are handled by the Patient Insurance Centre (PVK), and complications treated in private or public healthcare are not reported in the registers. The exclusion of PVK was a deliberate decision, as the focus of this study was on regulatory authorities that are directly involved in oversight and enforcement rather than those that handle compensation claims.

## Results

The study consisted of 25 complaints of 27 dermal filler treatment complications which are described in Table 1. KKV received 76% of the complaints, AVI 16%, and TUKES 8%. Valvira had no complaints to report on dermal fillers. In all cases, the reason for contacting the authorities was a complaint of unsuccessful treatment with dermal fillers. Since all identifying information was unavailable, sex or age distribution of the reporters was not available.

**Table 1:** Characteristics of complaints to Finnish Authorities of dermal filler treatment complications.

Finnish authority	KKV	TUKES	AVI	All
Number of complications	*n* = 20*	*n* = 3*	*n* = 4	*n* = 27
Number of complaints	*n* = 19	*n* = 2	*n* = 4	*n* = 25
**Facial region**				
Tear trough	3 (15%)	-	-	3 (11%)
Cheek	-	-	1 (25%)	1 (4%)
Lip	13 (65%)	2 (67%)	1 (25%)	16 (59%)
Chin, jawline	3 (15%)	1 (33%)	1 (25%)	5 (19%)
Unknown	1 (5%)	-	1 (25%)	2 (7%)
Altogether	20 (100%)	3 (100%)	4 (100%)	27 (100%)
**Type of reported complication**				
Nodule	11 (55%)	1 (33%)	-	12 (44%)
Unsuccessful treatment	4 (20%)	-	3 (75%)	7 (26%)
Migration	4 (20%)	-	-	4 (15%)
Infection	-	-	1 (25%)	1 (4%)
Discoloration	1 (5%)	-	-	1 (4%)
Numbness	-	1 (33%)	-	1 (4%)
Trismus	-	1 (33%)	-	1 (4%)
**Treatment with hyaluronidase**				
Have been treated	10 (50%)	-	-	10 (37%)
Going to treatment	3 (15%)	-	-	3 (11%)
Have been suggested for treatment	2 (10%)	-	-	2 (7%)
No treatment with hyaluronidase	5 (25%)	3 (100%)	4 (100%)	12 (44%)
**Registered industry/health care sector**				
Beauty salon	10 (53%)	2 (100%)	2 (50%)	14 (56%)
Medical clinic services	6 (32%)	-	2 (50%)	8 (32%)
Other healthcare services	1 (5%)	-	-	1 (4%)
Unknown	2 (11%)	-	-	2 (8%)
Altogether	19 (100%)	2 (100%)	4 (100%)	25 (100%)
**Qualifications of the practitioner**				
Medical doctor	1 (5%)	-	1 (25%)	2 (8%)
Nurse	6 (32%)	-	-	6 (24%)
Beauty salon employee	7 (37%)	2 (100%)	2 (50%)	11 (44%)
Unknown	5 (26%)	-	1 (25%)	6 (24%)
Altogether	19 (100%)	2 (100%)	4 (100%)	25 (100%)
**Year of complication occurrence**				
2024	5 (26%)	-	-	5 (20%)
2023	8 (42%)	-	1 (25%)	9 (36%)
2022	2 (11%)	1 (50%)	1 (25%)	4 (16%)
2021	4 (21%)	1 (50%)	-	5 (20%)
2018	-	-	1 (25%)	1 (4%)
2017	-	-	1 (25%)	1 (4%)
Altogether	19 (100%)	2 (100%)	4 (100%)	25 (100%)
**Situation of the complication in question at the time of complaints**				
Present	12 (63%)	2 (100%)	4 (100%)	18 (72%)
Non-present	7 (37%)	-	-	7 (28%)
Altogether	19 (100%)	2 (100%)	4 (100%)	25 (100%)

KKV: Finnish Competition and Consumer Authority; TUKES: Finnish Safety and Chemicals Agency; AVI: Regional State Administrative Agency.

* Two persons reported two complications in the same complaint.

**Figure 1:**
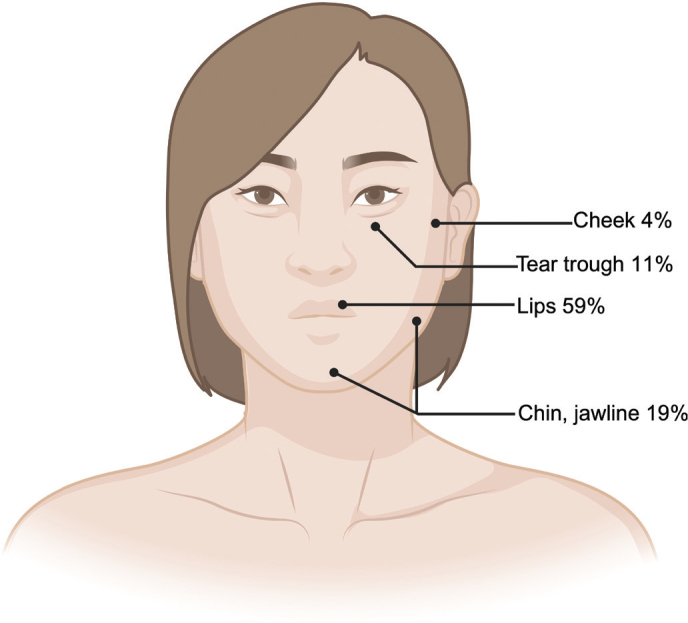
The facial areas affected by dermal filler treatment complications. In four complaints, the area was face, not otherwise specified (7%). Created with Biorender.com.

Lip was the most common region for dermal filler treatment complication since 59% of the complaints considered treatments on lips (Figure 1). The second most common was the chin on the jaw line (19%), followed by tear trough (11%). The exact filler product was not available from the records but in Finland, we expect it to be hyaluronic acid gels as the companies involved offered dermal filler treatments with hyaluronic acid. The most reported complication type was nodules (44%). According to the complaints, 56% of the complications occurred in beauty salons. The treatment had been received in medical clinics in 32% of the cases. The treatments in question were delivered by a beauty salon employee in 44% of the cases followed by a nurse in 24% of the cases. In two cases (8%), the complaint of the treatment was on a medical doctor. Among the reported complications, 37% were managed with hyaluronidase.

**Figure 2:**
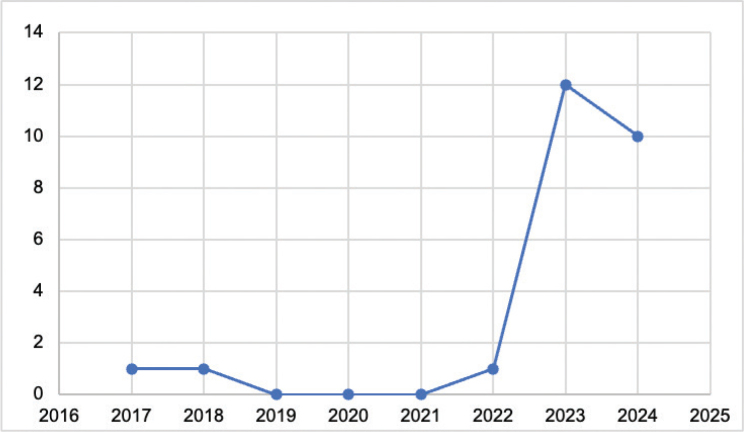
Number of complaints to Finnish Authorities of dermal filler treatment complications between the years 2017 and 2024.

The number of complaints increased after the year 2021. In this 7-year time period, 72% of the complications occurred in the last 3 years (Figure 2). Altogether, 72% of the reporting consumers stated that the complication is present at the time of their complaint.

## Discussion

Since dermal filler treatments are a quick way to diminish facial lines, their popularity has been risen [1], which reflects the increasing number of complications [13]. Young women are the main consumer groups for dermal filler treatments [18]. Before the year 2025, 25 complaints of 27 complications of dermal fillers were reported to Finnish authorities. In the complaints, the most common complication associated with dermal fillers was the formation of nodules in the lips. These 25 complaints most likely are only a peak of an iceberg, and in many cases, customers probably complain directly to performing practitioner. In Finland, there is a lack of government approved registry for unsuccessful esthetic treatment such as Save Face-registry in the UK [19]. However, even with this rather low number of complaints to the authorities in a country of 5.3 million inhabitants, there is a clear rise in number in the recent years.

In the UK with 69 million inhabitants, Save Face’s Consumer Complaints Audit Report of 2018 received 934 complaints, of which 83% of complications were carried out by non-medicinal practitioners such as beauticians, hairdressers, and laypersons which is similar to our study [19]. According to a systematic review by Tripathi et al., unexperienced practitioners have a higher risk of causing a vascular occlusion with filler treatments, which can lead to tissue necrosis and thus potential blindness [20]. The first death caused by a filler treatment was reported in the UK in 2024 [21]. In Finland, anyone can provide hyaluronic acid injection treatments. In our study, majority of treatments were performed by beauty salon employees with no healthcare background. Fortunately, the complications were minor. To our knowledge, in Finland, death caused by a filler treatment has not been reported. However, in spring 2024, a Finnish Broadcasting company YLE reported a case of cheek filler treatment performed by a cosmetologist which caused an infection requiring hospitalization [22].

Finnish legislation on dermal filler treatment differs from other Nordic countries, for example, Sweden and Norway, where dermal filler applications require training in healthcare [12, 23]. The Swedish National Board of Health and Welfare describes that esthetic treatments require medical competence, as treatment can pose significant health risks [24]. For this reason, Sweden in 2021 and Norway in 2022 tightened their legislations concerning aesthetic treatments and only licensed physicians, dentists, and nurses are permitted to perform aesthetic injection treatments [14, 15]. In addition, these procedures are prohibited for individuals under 18 years of age, unless there is a medical indication. In Finland, the administration of dermal filler treatments is not subject to age restrictions.

In the current study, most complications were reported to the Consumer and Competition Authority, KVV. This further indicates that most of filler treatment providers lacked healthcare training. In Finland, for healthcare professionals, it is mandatory to obtain patient insurance, enabling application of patient and consumer protection laws in cases of complications. Patient injury claims are handled by the Patient Insurance Centre. In this study, no information was requested from the Patient Insurance Centre since consumer complaints are not available from the current non-healthcare providers of filler treatments, who do not constitute patient record entries. If the filler treatment provider does not possess patient insurance, the client is considered as a consumer, not a patient, in the event of complications. [12, 23, 25, 26]. It seems rational that more complications from filler treatments have been occurred in Finland than the consumers have reported to the authorities. In the literature, most of the data are based on passive reporting systems, such as the Food and Drug Administration Manufacturer and User Facility Device Experience (FDA MAUDE), which likely leads to systematic underreporting [27, 28]. Both retrospective analyses and consensus articles highlight significant underreporting [29, 30]. Complications from filler treatments might be left unidentified and since cannot be reported [11]. It is possible that the practitioners might be reluctant to report complications due to potential reputational damage or legal issues [31]. Individuals who experience complications might feel shame or guilt, which may prevent them from reporting the incident [32].

Naming different filler types in the current study is not possible but in Finland, we expect them to be hyaluronic acid gels as the companies involved offered dermal filler treatments with hyaluronic acid. To perform botulinum toxin treatments in Finland, it requires healthcare training and a permit from Valvira since 2020. Therefore, only a medical doctor/dentist or a nurse working under a doctor’s supervision is permitted to provide these treatments. The service provider and service unit must be registered in the national service provider registry (Soteri) [33]. It is contradictory that only botulinum treatments were regulated, considering that potential complications related to the treatments are relatively mild [34]. Complications from hyaluronic acid gel treatments may require immediate first aid [6, 35], which is why they should be classified as procedures requiring advanced medical expertise, like botulinum treatments. According to this, Finnish authorities could regulate filler treatments similarly to Sweden, Denmark, and Norway, where filler treatments are classified as a procedure that requires advanced medical expertise. Then, the administration of filler treatments would be considered a healthcare activity, even when it is provided purely for aesthetic purposes. In addition, appropriate patient record entries would be required for the provided procedure. In case of filler treatment complication, this would allow their quicker management by aiding recognition and differential diagnosis [36]. Furthermore, age limitation would be advisable as in Sweden.

Strength of the study is that all public registries open for a consumers reporting were included in the study. As a limitation, we only searched public registries for complaints identified by the clients themselves and not by a healthcare professional. In Finland, dermal filler treatments are not restricted to healthcare professionals and it is likely that more complications have occurred. It seems that underreporting by clients and treatment performer distort complication rates, as Finnish legislation does not mandate adverse event reporting to a public registry. Complications that fall under patient insurance are handled by the Patient Insurance Centre, and complications treated in private or public healthcare are not reported in the registers used in the current study.

## Conclusions

In Finland, complaints made by individuals to authorities regarding complications from filler treatments are on the rise. The majority of these filler treatments were performed by individuals other than healthcare professional in beauty salon. It is important to note that filler treatments can carry serious health risks that require immediate first aid from a medical doctor or a dentist familiar with aesthetic medicine. Therefore, it would be advisable to consider limiting filler treatments to healthcare professionals only.

## Data Availability

All data generated or analyzed during this study are included in this published article.
